# Bacteriocins, a New Generation of Sustainable Alternatives to Antibacterial Agents in Primary Food Production Systems

**DOI:** 10.3390/molecules31020356

**Published:** 2026-01-19

**Authors:** Besarion Meskhi, Svetoslav Dimitrov Todorov, Dmitry Rudoy, Anastasiya Olshevskaya, Victoria Shevchenko, Tatiana Maltseva, Arkady Mirzoyan, Denis Kozyrev, Mary Odabashyan, Svetlana Teplyakova, Maria Mazanko

**Affiliations:** 1 Agribusiness Faculty, Don State Technical University, Gagarin Square 1, Rostov-on-Don 344000, Russia; 2 ProBacLab, Laboratório de Microbiologia de Alimentos, Departamento de Alimentos e Nutrição Experimental, Faculdade de Ciências Farmacêuticas, Universidade de São Paulo, São Paulo 05508-000, Brazil; 3 CISAS—Center for Research and Development in Agrifood Systems and Sustainability, Instituto Politécnico de Viana do Castelo, 4900-347 Viana do Castelo, Portugal

**Keywords:** aquaculture, agriculture, bacteriocins, antagonistic activity, crop production, livestock farming, poultry farming

## Abstract

Modern agriculture faces the critical need to develop sustainable, safe, and effective strategies for enhancing productivity, protecting plants and animals, and ensuring food security. Challenges posed by antibiotic resistance and the adverse environmental and consumer health impacts of chemical agents are driving the search for eco-friendly alternatives. In this context, bacteriocins—naturally occurring antimicrobial peptides synthesized by diverse bacteria—represent a promising alternative to traditional chemical compounds. This article reviews the potential and current advances in bacteriocin applications across agricultural sectors, with particular focus on their targeted antagonistic activity, structural diversity, commercial bacteriocin-based products, and their utilization in livestock farming, crop production, poultry farming, and aquaculture. Key findings demonstrate that bacteriocins, particularly nisin and pediocin PA-1, exhibit potent activity against major agricultural pathogens including *Listeria monocytogenes*, *Staphylococcus aureus*, *Clostridium perfringens*, and *Escherichia coli*, with efficacy rates reaching 90% in mastitis treatment and significantly reducing pathogen loads in poultry and aquaculture systems. Commercial products such as Nisaplin, Wipe Out, and ALTA 2431 have been successfully implemented in veterinary medicine and food production. In aquaculture, bacteriocins effectively control *Lactococcus garvieae*, *Aeromonas* spp., *Vibrio* spp., and *Pseudomonas aeruginosa*, contributing to sustainable disease management with minimal environmental impact. It can be suggested that bacteriocins may play an essential role in combating pathogens and offer viable alternatives to conventional antibiotics across primary food production systems, though optimization of production methods and regulatory frameworks remains essential for broader commercial adoption.

## 1. Introduction

When Fleming discovered penicillin, the scientific community believed it would be a silver bullet against infectious diseases. Antibiotics were used therapeutically, as well as preventively and even as growth promoters. However, soon after, reports of antibiotic resistance emerged. Prolonged and uncontrolled use of antibiotics has led to the development of resistance in both pathogenic microorganisms and commensal bacteria, including probiotic strains. By 2000, the World Health Organization (WHO) recognized this as a critical global health issue and recommended a gradual phase-out of antibiotics as feed additives (used for prevention or growth promotion) in livestock. This prompted the development of environmentally safer agents to combat pathogens [1], as agriculture required alternatives.

Bacteria employ diverse defense mechanisms, including a repertoire of non-ribosomally synthesized antibiotic-like metabolites, lytic enzymes, metabolic byproducts, protein exotoxins, and ribosomally produced antimicrobial peptides (bacteriocins) [2,3]. The first bacteriocin, colicin V, was reported by André Gratia in 1925 as a proteinaceous compound produced by *E. coli* [4], which was further classified as a microcin [5] due to its molecular weight being below 10 kDa. Later, Rogers and Whittier identified nisin, produced by a strain of the Gram-positive bacterium *Lactococcus lactis*, in 1928 [6]. Moreover, Jacob et al. [7] coined the term “bacteriocin” in 1953. In 1969, the Food and Agriculture Organization (FAO) and WHO approved nisin for food use [8]. Nisin subsequently received approval from the U.S. Food and Drug Administration (FDA) and the European Food Safety Authority (EFSA) and is now permitted in more than 48 countries [9,10]. According to U.S. FDA regulatory documents, nisin (commercialized as Nisaplin^®^) is allowed at a maximum of 2.9 mg per person per day [7]. It is designated as food additive E234 [11] by the European Union and by the British Standards Institution in the UK [12]. Additionally, pediocin PA-1 produced by *Pediococcus acidilactici*, is commercialized as the U.S. FDA-approved ALTA™ 2431. Furthermore, Micocin^®^, containing three bacteriocins (carnocyclin A, carnobacteriocin BM1, and piscicolin 126), has been approved for commercial applications [13].

Bacteriocins are of considerable interest as a foundation for next-generation therapeutics with promising applications in both human and veterinary healthcare, as well as the food industry [14]. Due to bacteriocins’ potent antibacterial properties and potential as natural preservatives [15], therapeutic agents, and agricultural tools, research on bacteriocins is rapidly expanding, with new producing strains, diverse bacteriocins, and novel applications continuously reported [7]. Furthermore, their efficacy against multidrug-resistant microbes has generated global interest in pharmaceutical applications, with particular focus on human, veterinary, and agricultural practices [12].

The diversity of delivery formats—such as purified concentrates, fermentates, and ready-to-use sanitizers—highlights the versatility of bacteriocins across various application environments. This adaptability enables their use not only as food preservatives but also as biocontrol agents against pathogens in agriculture and veterinary medicine.

## 2. Bacteriocins: Classification, Sources, and Mechanisms of Action

Bacteriocins are short protein molecules and peptides that exert antagonistic effects on other bacterial cells. It is generally accepted that virtually all bacteria produce at least one bacteriocin [2]. Archaea also produce similar antimicrobial substances known as archaeocins [16].

A key distinguishing feature of bacteriocins is their ribosomal synthesis, which fundamentally differentiates them from non-ribosomally synthesized peptides. The most critical characteristic is the presence of a specialized, genetically encoded immunity system in the producing strain that prevents self-destruction. Moreover, bacteriocins generally exhibit a narrow spectrum of activity, targeting phylogenetically related organisms, in contrast to broad-spectrum classical antibiotics.

The antagonistic activity of bacteriocins is characterized by a relatively narrow spectrum, typically directed against closely related bacterial species, reflecting their ecological role in niche competition. This targeted action offers a significant advantage by suppressing specific pathogens while minimizing disruption to the broader commensal microbiota.

### 2.1. Classification of Bacteriocins

The considerable structural and functional diversity of bacteriocins has led to the development of several classification systems. The most widely accepted and fundamental scheme, especially for bacteriocins from Gram-positive bacteria such as lactic acid bacteria (LAB), is based on their biochemical and genetic characteristics and classifies them into three main classes [17].

Class I comprises lantibiotics. These are small (<5 kDa), heat-stable peptides that undergo extensive post-translational modifications. A key feature is the presence of unusual amino acids, such as lanthionine and methyllanthionine, which form characteristic ring structures essential for their activity. The most prominent example is nisin.

Class II includes non-lantibiotic peptides. It encompasses small (<10 kDa), heat-stable peptides that lack extensive post-translational modifications. Several subclasses exist within this group. Subclass IIa consists of pediocin-like bacteriocins, noted for their potent anti-*Listeria* activity, with pediocin PA-1 as a key representative. Subclass IIb contains two-peptide bacteriocins, requiring two distinct peptides for optimal activity. Other single-peptide bacteriocins fall under Subclass IIc.

Class III consists of bacteriolysins. These are large (>30 kDa), heat-labile proteins whose antimicrobial effect arises not from pore formation in the membrane but from enzymatic degradation of the target cell wall, leading to cell lysis.

This three-class system provides a robust framework for understanding the primary modes of action and biosynthetic pathways of the most extensively studied bacteriocins.

Since bacteriocins constitute a highly heterogeneous group of compounds, this has led to the development of numerous classification systems that go beyond the three-class scheme outlined above. These alternative approaches are based on genetic and biochemical characteristics, as well as physicochemical properties such as molecular weight, thermostability, and resistance to proteolytic enzymes [18,19]. For example, some researchers have proposed classification into four distinct groups [11]. Another widely used classification for LAB bacteriocins divides them into two main classes: Class I (peptides with post-translational modifications) and Class II (peptides without significant structural alterations, permitting only limited modifications such as disulfide bridge formation) [20].

The evolution of these systems underscores the complexity of bacteriocin taxonomy. As data on chemical structures and modes of action have accumulated, some classifications have been revised. For instance, compounds initially assigned to a separate “Class IV” were subsequently reclassified after it was discovered that their antimicrobial activity is due to lytic action; they are now consistently included in Class III (bacteriolysins) of the primary classification system [21].

### 2.2. Sources of Bacteriocins

The most common and studied bacteriocins of Gram-negative bacteria are primarily colicins and microcins, with other types like pyocins also notable.

Colicins are high-molecular-weight bacteriocins (20–90 kDa) produced mainly by *E. coli*. Their mechanisms of action include forming pores in target bacterial membranes but they may have nuclease activity (DNase or RNase). Colicins are usually plasmid-encoded and lack post-translational modifications, released by producer cell lysis under stress via the SOS response. They target closely related Gram-negative bacteria by binding specific outer membrane receptors and using Tol or TonB systems for entry.

Microcins are small ribosomally synthesized peptides (10–20 kDa), heat-stable and encoded on chromosomes or plasmids. They often undergo proteolytic cleavage and post-translational modifications that enhance stability and effectiveness. They resemble antibiotics in mechanisms, sometimes sharing receptors and crossing pathways in bacterial membranes [22,23,24].

Bacteriocins of Gram-positive bacteria exhibit specific self-immunity mechanisms in producer strains and display varying antibacterial spectra, from narrow to broad, mostly targeting Gram-positive competitors.

The most common and studied bacteriocins of Gram-positive bacteria fall mainly into two major classes: lantibiotics (Class I) and non-lantibiotics (Class II). These ribosomally synthesized peptides typically contain 30 to 60 amino acids and exhibit a range of antibacterial spectra against Gram-positive bacteria. They are often heat-stable and produced with self-protection mechanisms by the producing strain.

Lantibiotics are characterized by post-translational modifications that form unusual amino acids such as lanthionine and beta-methyl lanthionine through dehydration and thioether ring formation. Lantibiotics have more complex structures with various heterocycles and modifications that influence their activity and specificity. They act mainly by targeting bacterial cell walls or membranes, with mechanisms including pore formation or inhibition of cell wall synthesis.

Non-lantibiotic bacteriocins are smaller peptides and are generally not extensively post-translationally modified like lantibiotics. They mainly target the membrane integrity of susceptible bacteria.

Many bacteriocins are produced by LAB, making LAB bacteriocins a major focus of study due to their potential applications in food preservation and antimicrobial therapy. A well-known example is nisin (lantibiotic), produced by LAB, which is widely studied for its potent antimicrobial activity mainly against closely related Gram-positive strains and is used as a food preservative [25,26].

One of the promising sources of bacteriocins for applications in agriculture, animal husbandry, and the food industry is *Bacillus* species.

In addition to bacteriocins, members of the genus *Bacillus* are prolific producers of a broad spectrum of antimicrobial agents [27], including peptide and lipopeptide antibiotics. This biosynthetic capacity, combined with their ability to form endospores, provides *Bacillus* strains with a significant competitive advantage, facilitating adaptation and survival in diverse ecological niches.

For example, the strain *Bacillus amyloliquefaciens* LBM 5006 produces a thermostable, low-molecular-weight peptide (5 kDa) active against spoilage and foodborne pathogenic bacteria such as *Listeria monocytogenes* and *Bacillus cereus*. Bacteriocins from *Bacillus cereus* (e.g., cerein 8A) disrupt the membrane integrity of *L. monocytogenes*. *Bacillus subtilis* is characterized by its ability to produce subtilosin. Common features of most *Bacillus* bacteriocins include high thermostability (withstand temperatures up to 121 °C), stability across extreme pH ranges, and resistance to proteolytic degradation. These properties make them promising candidates for applications in the food industry, agriculture, and medicine [28]. Notably, a high degree of antimicrobial peptide diversity can be observed even among different strains within the same bacterial species and genus (Table 1).

It should be noted that the ability to produce multiple bacteriocins with diverse physicochemical and functional properties is not limited to the genus *Bacillus* but is observed across various bacterial groups. This highlights the broad potential of bacteriocins as a source of diverse antimicrobial compounds.

### 2.3. Antimicrobial Activity Spectrum

The variety of mechanisms directly determines the spectrum of susceptible microorganisms, as summarized in Table 2.

Bacteriocins exhibit selective activity against Gram-positive pathogens, including *L. monocytogenes*, *Staphylococcus aureus*, *Bacillus cereus*, *Clostridium* spp., and representatives of enterococci, lactobacilli, lactococci, leuconostoc, and streptococci. For example, nisin, produced by *Lactococcus lactis* subsp. *lactis*, is active against a broad spectrum of *Listeria*, *Staphylococcus*, and other Gram-positive bacteria. In contrast, plantaricin C, expressed by *Lactiplantibacillus plantarum* LL441, inhibits the growth of *Staphylococcus carnosus* but shows no activity against *Listeria innocua*.

Certain bacteriocins, such as lactocin S (*Latilactobacillus sakei* L45) and thermophilin 13 (*Streptococcus thermophilus* SFi13), demonstrate activity against similar groups of microorganisms, exhibiting a narrow spectrum that includes only some strains of *L. monocytogenes* and *Bacillus* spp. Moreover, specific bacteriocins like bavaricin A (*Ltb. sakei* MI401) are ineffective against *Carnobacterium* spp. and *Brochothrix thermosphacta*, highlighting their narrow activity spectrum. Lacticin 3147 also demonstrates broad and potent antimicrobial potential against Gram-positive bacteria, with a spectrum comparable to that of nisin. Its lack of activity against Gram-negative bacteria confirms its specificity [52].

Of particular interest is pediocin PA-1 (*Pediococcus acidilactici* PAC 1.0), which not only suppresses the growth of *Listeria* and *Staphylococcus* species but also shows inhibitory effects against *Bacillus cereus* and *Clostridium* spp. This broad efficacy makes it a promising agent for applications in the food industry and agriculture. Conversely, piscicolin 126, produced by *Carnobacterium piscicola* JG126, is active against *Streptococcus* spp. and *Listeria* spp. but exhibits no activity against *Lactococcus* or *Staphylococcus* species.

Another bacteriocin of substantial interest, particularly in agriculture, is enterocin AS-48, produced by *Enterococcus faecalis* S-48 [53]. This cyclic, cationic, globular peptide is post-translationally modified by dedicated enzymes that ensure the head-to-tail amide bond linkage and demonstrates stability across a wide pH and temperature range [53]. Numerous studies have highlighted the particular potential of enterocin AS-48 for controlling pathogenic microorganisms in various food products, including meat, dairy, seafood, and plant-based matrices. Furthermore, enterocin AS-48 has been suggested for application against yeasts, molds, and other eukaryotic organisms. It exhibits antimicrobial effects against both Gram-positive and Gram-negative bacteria with varying efficacy, including representatives of the genera *Corynebacterium*, *Mycobacterium*, *Nocardia*, *Listeria*, *Lactobacilli*, *Lactococcus*, *Leuconostoc*, and *Pediococcus*. Moreover, enterocin AS-48 is effective against anaerobic and facultative anaerobic endospore-forming bacteria such as *Bacillus cereus*, *Bacillus coagulans*, *Bacillus subtilis*, *Bacillus licheniformis*, *Bacillus macroides*, members of the genus *Paenibacillus*, as well as the acidophilic species *Alicyclobacillus acidoterrestris* and *Alicyclobacillus acidocaldarius*, and *Geobacillus stearothermophilus*. Among strictly anaerobic endospore-forming bacteria, susceptibility to enterocin AS-48 has been observed in *Clostridium perfringens*, *Clostridium sporogenes*, and *Clostridium tetani* [54].

**Table 2:** Some examples for spectrum of antibacterial activity of bacteriocins [55,56,57].

Bacteriocin-Producing Strain	Bacteriocin	Susceptible Bacteria	Resistant Bacteria
*Lactococcus lactis* DPC3147	Lacticin 3147	*Enterococcus*, *Lactobacillus* ^1^, *Lactococcus*, *Leuconostoc*, *Pediococcus*, *Streptococcus*, *L. monocytogenes*, *Listeria innocua*, *Staphylococcus aureus*, *Bacillus*, *Clostridium*	– ^2^
*Latilactobacillus sakei* L45	Lactocin S	*Enterococcus*, *Lactobacillus*, *Lactococcus*, *Leuconostoc*, *Pediococcus*, *L. monocytogenes*, *Listeria innocua*, *Staphylococcus*, *Bacillus cereus*, *Clostridium*	–
*Lactococcus lactis* subsp. *lactis*	Nisin	*Enterococcus*, *Lactobacillus*, *Lactococcus*, *Leuconostoc*, *Pediococcus*, *L. monocytogenes*, *Listeria innocua*, *Listeria grayi*, *Listeria ivanovii*, *Listeria seeligeri*, *Listeria welchimeri*, *Staphylococcus*	–
*Lactobacillus plantarum* LL441	Plantaricin C	*Enterococcus*, *Lactobacillus*, *Lactococcus*, *Leuconostoc*, *Pediococcus*, *Streptococcus*, *Staphylococcus carnosus*, *Bacillus*, *Clostridium*	*Listeria innocua*
*Streptococcus thermophilus* SFi13	Thermophilin 13	*Enterococcus*, *Lactobacillus*, *Lactococcus*, *Leuconostoc*, *Streptococcus*, *L. monocytogenes*, *Listeria innocua*, *Staphylococcus carnosus*, *Bacillus*, *Clostridium*	–
*Lactobacillus acidophilus* TK9201	Acidocin A	*Enterococcus*, *Lactobacillus*, *Pediococcus*, *Streptococcus*, *L. monocytogenes*	*Bacillus subtilis*
*Lactobacillus sakei* MI401	Bavaricin A	*Enterococcus*, *Lactobacillus*, *Lactococcus*, *Leuconostoc*, *Pediococcus*, *L. monocytogenes*	*Carnobacterium*, *Streptococcus*, *Brochothrix thermosphacta*, *Bacillus*, *Staphylococcus*
*Lactobacillus curvatus* LTH1174	Curvacin A	*Carnobacterium*, *Enterococcus*, *Lactobacillus*, *Lactococcus*, *Pediococcus*, *L. monocytogenes*, *Listeria innocua*, *Listeria ivanovii*	*Leuconostoc*, *Clostridium*
*Carnobacterium divergens* V41	Divercin V41	*Enterococcus*, *Lactobacillus*, *Pediococcus*, *L. monocytogenes*, *Listeria innocua*, *Listeria ivanovii*	*Lactococcus*, *Leuconostoc*
*Enterococcus faecium* CTC492	Enterocin A	*Enterococcus*, *Lactobacillus*, *Pediococcus*, *L. monocytogenes*, *Listeria innocua*	*–*
*Lactococcus lactis* MMFII	Lactococcin MMFII	*Enterococcus*, *Lactobacillus*, *Lactococcus*, *Listeria ivanovi*	*–*
*Leuconostoc mesenteroides* Y105	Mesentericin Y105	*Enterococcus*, *Lactobacillus*, *Leuconostoc*, *Pediococcus*, *L. monocytogenes*, *Listeria innocua*, *Listeria ivanovi*	*Lactococcus*
*Enterococcus mundtii* ATO6	Mundticin	*Carnobacterium*, *Enterococcus*, *Lactobacillus*, *Leuconostoc*, *Pediococcus*, *L. monocytogenes*, *Listeria innocua*	*–*
*Pediococcus acidilactici* PAC 1.0	Pediocin PA-1	*Carnobacterium*, *Enterococcus*, *Lactobacillus*, *Lactococcus*, *Leuconostoc*, *Pediococcus*, *L. monocytogenes*, *Listeria innocua*, *Listeria ivanovii*, *Staphylococcus*, *Bacillus cereus*, *Clostridium*	*–*
*Carnobacterium piscicola* V1	Piscicocin V1a	*Carnobacterium*, *Enterococcus*, *Lactobacillus*, *Leuconostoc*, *Pediococcus*, *L. monocytogenes*, *Listeria innocua*	*Lactococcus*, *Bacillus cereus*, *Clostridium*, *Staphylococcus aureus*
*Carnobacterium piscicola* V1	Piscicocin V1b	*Carnobacterium*, *Enterococcus*, *Lactobacillus*, *Leuconostoc*, *Pediococcus*, *L. monocytogenes*, *Listeria innocua*	*Lactococcus*, *Bacillus cereus*, *Clostridium*, *Staphylococcus aureus*
*Carnobacterium piscicola* JG126	Piscicolin 126	*Carnobacterium*, *Enterococcus*, *Lactobacillus*, *Leuconostoc*, *Pediococcus*, *Streptococcus*, *L. monocytogenes*, *Listeria grayi*, *Listeria ivanovii*, *Listeria seeligeri*, *Bacillus thermosphacta*	*Bacillus*, *Clostridium*, *Lactococcus*, *Leucobacter denitrificans*, *Staphylococcus*
*Lactobacillus sakei* LB706	Sakacin A	*Enterococcus*, *Lactobacillus*, *Pediococcus*, *L. monocytogenes*, *Listeria innocua*, *Listeria ivanovii*	*Lactococcus*, *Leuconostoc*
*Lactobacillus sakei* LB674	Sakacin P	*Enterococcus*, *Lactobacillus*, *Pediococcus*, *L. monocytogenes*, *Listeria innocua*, *Listeria ivanovii*	*Lactococcus*, *Leuconostoc*
*Streptococcus bovis* HC5	Bovicin HC5, nisin	*Alicyclobacillus acidoterrestris*	*–*
*Enterococcus faecium* MMRA	Enterococin A	*L. monocytogenes*	*–*
*Lactococcus lactis* CGMCC20699	Bacteriocin RSQ04	*L. monocytogenes*	*–*
*Pseudomonas aeruginosa* QDD1	Pyocin QDD1	*Staphylococcus aureus*, *Bacillus cereus*	*–*
*Lactobacillus plantarum LSB1*	LSB1	*Staphylococcus argenteus*	*–*
*Lactobacillus delbrueckii*	ET05, ET12, ET32, ET34	*L. monocytogenes*	*–*
*Lactobacillus curvatus*	MBSa2, MBSa3	*L. monocytogenes*	*–*

^1^—the term “*Lactobacillus*” was used as the taxonomical name before changes in the taxonomy of lactobacilli in 2020 suggested by Zheng et al. [58]; ^2^—“–” indicates no data available. The nomenclature of the producer strains has been updated and abbreviated in accordance with the recent taxonomic revisions outlined in Note 1, including those based on the work by Todorov et al. [59].

A notable characteristic of certain bacteriocins is their capacity to inhibit viral pathogen transmission and replication by interfering with glycoprotein synthesis during the late stages of viral assembly. This property suggests a novel potential application for bacteriocin-producing probiotic strains as antiviral therapeutics. Bacteriocins derived from probiotic bacteria may modulate the immune system and enhance host resistance against viral agents such as COVID-19 [60]. Todorov et al. [61] reported that *Enterococcus mundtii* ST4V produces a bacteriocin with a molecular mass of 3.95 kDa exhibiting activity against various Gram-positive and Gram-negative pathogens of food and clinical relevance. Furthermore, this antimicrobial peptide demonstrated pronounced antiviral activity, leading to inactivation of herpes simplex viruses HSV-1 and HSV-2, poliovirus PV3, and measles virus, with viral inhibition efficacy of 95.5–99.9% against HSV-1, HSV-2, and measles virus. Importantly, this peptide showed no cytotoxicity against Vero cells even at fourfold higher concentrations, confirming the specificity of its antiviral effect [61]. Subsequent studies have also reported antiviral effects of bacteriocins [62,63,64].

### 2.4. Mechanism of Action

The antagonistic activity of bacteriocins is characterized by a relatively narrow spectrum, typically directed against closely related bacterial species, reflecting their ecological role in niche competition. This targeted action offers a significant advantage by suppressing specific pathogens while minimizing disruption to the broader commensal microbiota. Bacteriocins exhibit antagonistic effects on susceptible target cells through diverse and specific modes of action. Although detailed structure–antibacterial activity relationships are known for only a limited number of bacteriocins, studies confirm their ability to disrupt key cellular structures. Various classes of bacteriocins selectively interact with cell wall components, compromise membrane integrity, inhibit nucleic acid synthesis, or suppress enzyme activity. This molecular versatility underlies their broad antimicrobial activity and biotechnological potential [7].

The most common mechanism, characteristic of many Class I and Class II bacteriocins, involves disruption of cytoplasmic membrane integrity. Cationic and amphipathic bacteriocins such as nisin (Class I) and pediocin PA-1 (Class IIa) initially bind to specific receptor molecules in cell wall-lipid II for nisin and the mannose–phosphotransferase system (Man-PTS) for pediocin PA-1. After binding, the peptides insert into the lipid bilayer, orienting their hydrophobic regions. Oligomerization of bacteriocin molecules leads to the formation of transmembrane pores, triggering rapid efflux of vital ions (K^+^), metabolites, and ATP, membrane depolarization, and ultimately collapse of the proton motive force, resulting in cell death [65,66]. This mechanism explains the high efficacy of these bacteriocins against pathogens such as *L. monocytogenes* and *Staphylococcus aureus*.

Certain bacteriocins can penetrate the cell and target intracellular components. For example, some bacteriocins (such as colicins produced by Gram-negative bacteria) exhibit DNase or RNase activity, leading to genetic material degradation [67]. Others inhibit essential enzymes. Although less common, this mechanism highlights the strategic diversity of bacteriocins in microbial competition. For instance, pore-forming bacteriocins (nisin, pediocin PA-1) act against a broad range of Gram-positive bacteria, while bacteriolysins and bacteriocins with intracellular targets often display a narrow, highly specific spectrum. Combining different mechanisms of action within a single preparation or producer strain represents a promising strategy to counteract potential resistance development.

Accumulated data on their mechanisms of action facilitate the expansion of their application scope [68]. For example, bacteriocins have been proposed for treating peptic ulcer disease by inhibiting *Helicobacter pylori*; for cancer therapy by inhibiting DNA replication and membrane protein synthesis, thus inducing apoptosis or cytotoxicity in tumor cells; for the treatment and prevention of facial skin diseases; and as plant growth stimulants [69].

## 3. Applications of Bacteriocins

### 3.1. Bacteriocins in Animal Husbandry and Poultry Farming

In animal husbandry, antibiotics have been extensively used for three primary purposes: (A) treating established infections, (B) preventing infectious diseases, and (C) promoting animal growth. Although many countries have banned the use of antibiotics in feed for disease prevention and growth promotion, they remain available over the counter in some nations (e.g., Australia, Canada, China, USA). This practice exposes bacteria to sublethal doses of antibiotics, facilitating the development of resistance. It is noteworthy that the use of antibiotics for growth promotion in animals is based on observational findings published over 50 years ago [1].

Due to their unique properties, bacteriocins find applications in various agricultural sectors, including animal husbandry. Numerous bacteriocins, such as nisin U, uberolysin, carnobacteriocins, and enterocin RM6, demonstrate high activity against mastitis pathogens (*Staphylococcus aureus*, *Streptococcus agalactiae*) and other harmful bacteria (including *L. monocytogenes* and *Enterobacteriaceae*), significantly reducing their microbial load both in vitro and in vivo.

Specifically, for bovine mastitis, a nisin-based disinfectant wipe product, Wipe Out^®^ (Immucell, Portland, ME, USA), has been approved. For intramammary infusion, the preparation Mast Out^®^, also containing nisin as the active ingredient, shows promise. Nisin is the most extensively studied bacteriocin for mastitis therapy, with its efficacy confirmed both in vitro and in vivo. Studies have shown that nisin can achieve up to 90% efficacy in eliminating pathogens, including resistant strains of *Staphylococcus aureus*. Specifically, intraperitoneal administration of nisin achieved a 90.1% cure rate for subclinical mastitis caused by *Streptococcus agalactiae*. Even low concentrations of nisin (19.5 IU/mL) inhibit the growth of key pathogens such as *Streptococcus uberis*, *Streptococcus agalactiae*, and *Streptococcus dysgalactiae*.

Beyond nisin, other bacteriocins such as lacticin 3147 are under investigation. Teat dipping with a solution containing this bacteriocin resulted in the elimination of up to 97% of *Streptococcus agalactiae* strains. Bacteriocins morricin 269 and kenacin 404, produced by different strains of *Bacillus thuringiensis*, have demonstrated in vitro activity against a broad spectrum of mastitis pathogens. These findings highlight the high potential of bacteriocins as alternatives to conventional antibiotics in mastitis management [70].

Experimental trials treating clinical mastitis in lactating dairy cows included a comparative analysis of nisin efficacy versus the standard antibiotic gentamicin. The study found that nisin achieved a cure rate for mastitis comparable to gentamicin. The clinical efficacy of nisin and gentamicin was similar, at 90.2% and 91.1%, respectively. Furthermore, nisin is rapidly cleared from milk; after 12 h, its concentration is 4.5 IU/mL, which is significantly below the permissible limit of 500 mg/mL established in China [71].

Experimental studies have demonstrated that treatment with lacticin 3147 significantly reduced pathogen counts, with *Staphylococcus aureus* decreasing by 80%, *Streptococcus dysgalactiae* by 97%, and *Streptococcus uberis* by 90%. Additionally, lacticin NK34 provided 80% survival in mice challenged with lethal doses of staphylococci, underscoring the potential of bacteriocins in combating infections, including mastitis.

The establishment of a stable microbiome in broilers is a stepwise process of microbial colonization that results in a resilient community conferring pathogen resistance through competitive exclusion. However, in industrial poultry farming, this process is frequently disrupted due to impaired development of a balanced microbial community. Numerous strains of autochthonous gastrointestinal tract (GIT) microflora in broilers exhibit bacteriocinogenic activity, providing them with a colonization advantage. The application of purified bacteriocins as feed additives offers promising avenues for targeted modulation of the intestinal microbiome and enhanced activity of autochthonous bacteriocin-producing strains [72].

One of the most significant challenges in poultry farming is intestinal infection, which leads to increased flock mortality, substantial production losses, and the risk of contamination of raw materials intended for human consumption with dangerous pathogens. Within the poultry industry, the primary pathogens limiting the sector’s growth are the bacteria *Clostridium perfringens* and *E. coli*. *Clostridium perfringens*, an anaerobic bacterium colonizing the chicken intestine, causes necrotic enteritis. According to earlier estimates, this disease inflicted annual economic losses of 2 billion USD [73]. The increasing prevalence of resistant strains is driving the development of novel alternative strategies for managing the epizootic situation on poultry farms [1].

Necrotic enteritis in broilers is caused by netB-positive *Clostridium perfringens* type A strains, which exhibit enhanced suppression of other strains of this bacterium. This selective competitiveness promotes the dominance of toxigenic strains that damage the intestinal tract. In a study by Timbermont et al. [73], the bacteriocin perfrin was isolated from both healthy chickens and those with confirmed necrotic enteritis. Purification was performed using cation-exchange chromatography followed by hydrophobic interaction chromatography.

Among 50 analyzed avian *Clostridium perfringens* strains, 10 produced perfrin. Nine of these were isolated from birds with necrotic enteritis, while one originated from a healthy bird; all perfrin-positive strains were netB-positive. This observation led the authors to propose that perfrin may play a role in the pathogenesis of necrotic enteritis [73].

The efficacy of bacteriocins against *E. coli* was demonstrated in a study by Ogunbanwo et al. [74]. Broilers challenged with *E. coli* and administered bacteriocin via drinking water or receiving the probiotic *Lactiplantibacillus* plantarum F1 exhibited reduced clinical symptoms compared to the infected control group. Body weight and feed intake did not differ significantly between the treatment groups. Microbiological analysis confirmed the efficacy of both bacteriocin and probiotic, as significantly fewer *E. coli* isolates were recovered from the liver: 60% in the control group versus 8% in the bacteriocin group and 12% in the probiotic group. These results indicate that bacteriocin and probiotic treatments can effectively mitigate the consequences of *E. coli* infection in broilers [74].

To identify alternative means for controlling contamination of poultry with *Clostridium perfringens* and *L. monocytogenes*, Shin et al. [75] screened 291 bacterial strains isolated from broiler chicken gastrointestinal tracts for potential use as probiotics with broad-spectrum antimicrobial activity. Bacteriocins successfully isolated from strains *Enterococcus faecium* SH 528, *Enterococcus faecium* SH 632, and *Pediococcus pentosaceus* SH 740 effectively inhibited the growth of *Clostridium perfringens* and *L. monocytogenes*. The identified antimicrobial peptides retained activity after exposure to 60 °C for 30 min and were unaffected by organic dyes. The authors emphasize the potential application of these compounds in poultry farming to reduce bacterial infections [75].

Another significant zoonotic pathogen in poultry farming is *Campylobacter jejuni*, the causative agent of human gastroenteritis. The primary source of human infection is commercial broiler carcasses. Contamination levels exceeding 103 CFU/g correlate with consumer infection cases, whereas levels below 102 CFU/g do not lead to human illness. Reducing *Campylobacter* colonization in broilers pre-slaughter is a key strategy to minimize carcass contamination. In a review by Svetoch and Stern [76], based on extensive scientific literature analysis, a strategy utilizing bacteriocins to combat this infection was proposed. The authors concluded that administering bacteriocins via broiler drinking water effectively reduces *Campylobacter* colonization. Birds receiving bacteriocins during the final 3 days before slaughter exhibited a 105- to 106-fold reduction in cecal bacterial counts. This method is operationally simple and could significantly reduce the risk of human campylobacteriosis transmission via meat [76].

Bacteriocins, particularly nisin, exert potent antimicrobial effects. Existing evidence indicates that nisin can modulate the broiler gastrointestinal microbiota, suppress the proliferation of opportunistic pathogens (*Enterobacteriaceae*, *Clostridium perfringens*), and potentially enhance productivity. A study by Kierończyk et al. investigated the effects of salinomycin (60 mg/kg diet), nisin (2700 IU/kg diet), salinomycin (60 mg/kg diet) combined with nisin (2700 IU/kg diet), monensin (100 mg/kg diet), and monensin (100 mg/kg diet) combined with nisin (2700 IU/kg diet). Supplementing broiler diets with nisin reduced the feed conversion ratio (FCR) and increased body weight, positively impacting production economics. Furthermore, nisin supplementation had no adverse effects on tibia bone mineralization or blood biochemical parameters. These findings suggest that nisin holds potential as an effective growth-promoting feed additive without disrupting metabolic processes [77].

Due to its safety for humans and effectiveness against resistant bacteria, nisin warrants further investigation as a promising agent for animal treatment. Moreover, novel bacteriocins, such as sonorensin, inhibit not only bacterial pathogens (including resistant strains) but also fungal infections, broadening their potential applications in veterinary medicine and food safety [19].

Although the direct application of antiviral bacteriocins in agriculture has been less extensively studied than their antibacterial activity, this area holds significant promise for integrated biosecurity strategies within agro-industrial systems. The ability of bacteriocins and their producer probiotic strains to suppress viral pathogens may enable the development of novel protective agents for agricultural animals (e.g., against avian influenza virus and African swine fever virus). This approach could reduce reliance on traditional chemical agents and antibiotics.

### 3.2. Bacteriocins in Aquaculture

Aquaculture is a vital agricultural sector that has demonstrated dynamic growth in production volumes in recent years. According to some estimates, approximately 109 million tons of fish and seafood for human consumption will be produced under aquaculture conditions by 2030, while capture fisheries are projected to provide only 74 million tons [78]. High consumer demand for fish and seafood drives the intensification of production, which is associated with several challenges, among which bacterial diseases play a critical role.

While bacteriocins demonstrate significant potential for disease management in aquaculture, their practical implementation faces several key challenges that warrant careful consideration when interpreting the research findings presented below. Unlike terrestrial animal production systems where several bacteriocin-based products have achieved regulatory approval and commercial adoption, the aquaculture sector currently faces unique barriers. Critical factors affecting bacteriocin efficacy and commercial viability in aquatic environments include the following: (1) species-specific responses across diverse cultured organisms (finfish, crustaceans, mollusks); (2) variable water chemistry conditions (salinity, pH, temperature) affecting bacteriocin stability and activity; (3) rapid dilution effects in aquatic systems; (4) complex regulatory frameworks differing across major aquaculture-producing nations; and (5) cost-competitiveness compared to established antimicrobials and vaccines. Despite these limitations, accumulating evidence demonstrates the promise of bacteriocin-based interventions under controlled conditions, as illustrated by the following studies.

To address issues related to antibiotic resistance in aquaculture systems, bacteriocin-producing bacteria are currently widely applied. These bacteria are practical to implement and do not contribute to antibiotic resistance in cultivation systems. Similar to other agricultural sectors, bacteriocins represent promising eco-friendly alternatives to conventional antibacterial agents in aquaculture. The inhibitory activity of several well-characterized bacteriocins against relevant aquaculture pathogens has been confirmed experimentally, as summarized in Table 3. For example, enterocin AS-48, a circular peptide with a known structure and mode of action, exhibits efficacy against *Lactococcus garvieae* [53,54]. Likewise, mundticin KS and nisin Z, both members of established bacteriocin classes, have demonstrated activity against pathogens such as *Pseudomonas aeruginosa* and *Listeria garvieae*, respectively [61,79]. The use of these well-characterized molecules provides a clear and transferable strategy for disease control in aquaculture systems.

The application of bacteriocins in aquaculture systems is implemented through two primary strategies: the introduction of live bacteriocin-producing strains as probiotics or the direct use of purified bacteriocin preparations. When live producers are utilized, they are typically administered via feed or directly into the water. These strains may subsequently colonize the host’s gastrointestinal tract or the environment, enabling in situ bacteriocin production that competitively excludes pathogens. This approach depends on the viability and colonization efficacy of the strain. Alternatively, purified or semi-purified bacteriocins can be directly incorporated into feed pellets or introduced into the aquaculture system. This method ensures the delivery of a defined, active dose independent of bacterial viability and allows for rapid intervention. However, it may be more costly and face challenges related to stability in the aquatic environment. The choice between these strategies depends on factors such as the target pathogen, the cultivation system, cost-effectiveness, and regulatory considerations.

A broader range of bacterial isolates from aquatic and terrestrial environments has shown potential for biocontrol, although the antimicrobial agents involved are often poorly characterized. For example, strains of *Bacillus*, *Lactococcus lactis*, *Carnobacterium divergens*, and *Lactiplantibacillus plantarum* have been reported to inhibit fish pathogens such as *Aeromonas hydrophila*, *Vibrio parahaemolyticus*, and various *Vibrio* species [94,95,96,97,98]. In many cases, the activity is attributed to bacteriocins like sublancin, divercin, or bacteriocin FGC-12 [99], but these compounds may not be fully purified or structurally defined. Furthermore, other antimicrobials such as pyocins from *Pseudomonas* [100], the pigment pyocyanin [101], cyanobactins from cyanobacteria [102], and surfactin [103] also demonstrate antagonism against aquaculture pathogens. It is important to note that for some of these substances, the term “bacteriocin” is used provisionally, and their exact nature and mode of action require further elucidation. Despite this, the producing strains themselves hold promise as probiotics, provided they are sourced from non-pathogenic isolates and thoroughly evaluated for safety [104,105,106].

The use of microorganisms for disease control in aquaculture is rapidly gaining momentum across diverse cultured species [106]. Research has shown that the probiotic *Lactobacillus acidophilus* possesses antimicrobial properties, effectively protecting the shrimp *Penaeus monodon* against pathogenic microorganisms, including *Vibrio parahaemolyticus*, *Vibrio cholerae*, *Vibrio alginolyticus*, and *Vibrio harveyi* [107]. Bacteriocins secreted by various bacteria from diverse sources, including marine, freshwater, and terrestrial environments, are employed in fish farming systems. Surfactin, an antiviral compound produced by *Pseudomonas aeruginosa*, *Acinetobacter calcoaceticus*, and *Bacillus subtilis*, has been reported as effective in combating bacterial and viral diseases in aquaculture [103]. Thus, bacteriocins hold significant potential for sustainable health management in aquaculture, combining high specificity, safety, and minimal environmental impact.

Future developments in bacteriocin application in aquaculture are likely to focus on overcoming existing limitations and scaling up production. Key research priorities include optimization of cost-effective methods for both upstream production and downstream purification to ensure economic viability of large-scale manufacturing. Promising formulation technologies, such as microencapsulation, could enhance stability and enable targeted delivery of bacteriocins in feeds and aquatic environments. Furthermore, establishing clear regulatory frameworks for bacteriocin use in aquaculture will be crucial for commercial adoption and integration into aquaculture practices, ultimately contributing to safer aquatic food production systems.

#### Commercial Bacteriocin Products for Aquaculture Applications

Nisin-based applications represent the most extensively studied commercial bacteriocin in aquaculture contexts. Nisaplin (Danisco/DuPont, Copenhagen, Denmark), containing 2.5% pure nisin, has been evaluated for controlling pathogenic bacteria in various aquatic species. López et al. [108] demonstrated that nisin treatment significantly extended the shelf life of vacuum-packed rainbow trout (*Oncorhynchus mykiss*) fillets by inhibiting biogenic amine production and pathogen growth during refrigerated storage. Similarly, nisin combined with modified atmosphere packaging effectively maintained Atlantic salmon quality by reducing microbial loads of Gram-positive pathogens. More recently, Kazuń et al. [109] reported that nisin administration enhanced immune cell activity in the fish head kidney, demonstrating potential for in vivo disease prevention. Moroni et al. [110] further confirmed that nisin-producing *Lactococcus lactis* strains exhibit activity against major aquaculture pathogens including *Bacillus*, *Clostridium*, and *Listeria* species, positioning nisin as a viable therapeutic candidate for fish farming.

Probiotic-based bacteriocin delivery represents the most commercially viable strategy for aquaculture applications. Several commercial probiotic formulations contain bacteriocinogenic strains, although the bacteriocin activity is often not explicitly marketed as the primary mode of action. AquaStar^®^ Growout (Biomin Holding GmbH, Getzersdorf, Austria), a commercial probiotic mixture containing *Bacillus subtilis*, *Enterococcus faecium*, *Limosilactobacillus reuteri*, and *Pediococcus acidilactici*, has demonstrated enhanced antioxidative capacity and immunity in Nile tilapia (*Oreochromis niloticus*) and rainbow trout (*Oncorhynchus mykiss*). El-Kady et al. [111] confirmed that such commercial probiotics, when applied as water additives, significantly improved water quality, fish performance, blood biochemical parameters, and immunity in intensive aquaculture systems.

Pereira et al. [112] isolated bacteriocinogenic probiotic bacteria from aquatic environments, detecting genes encoding nisin, enterocin A, enterocin B, enterocin P, and mundticin KS. These strains demonstrated antimicrobial activity against major aquaculture pathogens and resistance to gastric acid and bile salts, suggesting their suitability for commercial probiotic development. Garcés et al. [97] further demonstrated that bacteriocin-producing *Lactobacilli* strains administered via feed supplementation effectively controlled *Aeromonas hydrophila* infections in fish, with bacteriocin production providing competitive advantages in the intestinal environment. A comprehensive review by Pereira et al. [112] documented multiple successful applications of bacteriocin-producing probiotics across diverse aquaculture species, including shrimp, tilapia, salmon, and seabass, with documented improvements in growth performance, feed conversion ratio, and lysozyme activity.

Enterocin AS-48 has emerged as a particularly promising candidate for aquaculture applications due to its broad-spectrum activity and stability. Rahayu et al. [113] reported that enterocin AS-48 administered via immersion or intraperitoneal injection effectively controlled lactococcosis in rainbow trout caused by *Lactococcus garvieae*, a major pathogen in salmonid aquaculture. The study demonstrated that semi-purified AS-48 produced from inexpensive food-based substrates could be applied as sustainable bath treatments, offering a practical and economically viable approach for commercial fish farms.

Micocin, a multi-bacteriocin preparation containing carnocyclin A, carnobacteriocin BM1, and piscicolin 126, holds particular relevance for aquaculture applications. Piscicolin 126, originally isolated from *Carnobacterium piscicola* JG126 (a fish-associated bacterium), exhibits inhibitory activity against *Listeria monocytogenes*, *Aeromonas salmonicida*, *Vibrio anguillarum*, and *Yersinia ruckeri*. Although primarily marketed for food preservation, Micocin’s origin from aquatic bacteria and documented efficacy against fish pathogens position it as a candidate for adapted aquaculture formulations.

Emerging bacteriocin products for shrimp aquaculture show particular commercial promise. Organicin Scientific has developed bacteriocin-incorporated feed specifically targeting *Vibrio* infections in farmed shrimp, demonstrating nearly 200% improvement in survival of shrimp challenged with Early Mortality Syndrome (EMS). The high thermostability of these bacteriocins enables their incorporation into feed during extrusion processing without loss of activity, facilitating straightforward adoption by farmers across recirculating aquaculture systems (RAS), pond, and offshore cage operations.

Antimicrobial packaging applications represent another emerging commercial opportunity. Bacteriocin-coated films have been successfully tested for extending the shelf life of processed seafood products. Studies demonstrated that low-density polyethylene films coated with nisin using methylcellulose as a carrier significantly reduced *Listeria monocytogenes* contamination on cold-smoked salmon. Similarly, bacteriocin-incorporated polyamide films extended the shelf life of fresh oysters from 9 to 12 days at 10 °C by inhibiting coliform and aerobic bacterial growth. Gumienna et al. [114] reported effective inhibition of *Listeria monocytogenes*, *Staphylococcus aureus*, *Bacillus cereus*, and *Alicyclobacillus acidoterrestris* in cold-smoked salmon packaged with bacteriocin-containing biodegradable polymers.

Regulatory and market barriers significantly constrain commercial bacteriocin product development for aquaculture. Key challenges include (1) the need for species-specific efficacy and safety data across diverse cultured organisms (finfish, crustaceans, mollusks); (2) variable water chemistry conditions (salinity, pH, temperature) affecting bacteriocin stability and activity; (3) complex regulatory frameworks differing across major aquaculture-producing nations; and (4) cost-competitiveness compared to conventional antimicrobials and vaccines.

### 3.3. Bacteriocin Applications in Crop Production Systems

Bacterial phytopathogens cause significant economic losses by inducing various plant diseases [115], disproportionately affecting food security in developing regions. Over 200 pathogenic species across more than 25 bacterial genera—including *Xanthomonas*, *Pseudomonas*, *Erwinia*, *Xylella*, *Ralstonia*, *Pectobacterium*, *Pantoea*, *Agrobacterium*, *Burkholderia*, *Acidovorax*, *Clavibacter*, and *Streptomyces*—trigger symptoms such as rot, cankers, galls, wilts, and leaf or fruit spotting. These infections compromise both crop yield and quality, presenting major agricultural challenges.

Effective control options remain limited due to the narrow spectrum of available chemicals. Conventional agents, such as antibiotics and copper-based formulations (e.g., Bordeaux mixture or copper-based nanoparticulate solutions like Kocide^®^ 3000, Pune, India), show efficacy but their application is strictly regulated due to environmental concerns—specifically soil bioaccumulation and the development of resistant strains. Chemical pollution in agriculture now represents a critical ecological threat [116]. While the chemical industry has historically provided and continues to offer effective solutions for controlling plant spoilage and pathogens, there is a growing demand for sustainable agricultural practices that reduce chemical inputs. This trend highlights the need for new approaches, with biological control strategies and natural antimicrobials receiving increasing research focus.

Bacteriocins are promising alternatives due to their broad-spectrum antimicrobial activity combined with minimal disruption to ecosystems. Beyond pathogen inhibition, emerging evidence indicates that bacteriocins also act as plant bio-stimulants, enhancing growth and stress tolerance [9]. Their targeted antimicrobial effects confer competitive advantages to producing strains by allowing niche displacement [22], while simultaneously reducing anthropogenic environmental impacts.

Soil harbors high bacterial diversity, with the rhizosphere being densely populated by numerous taxa—particularly Gram-positive bacteria, which typically reproduce more efficiently and have higher survival rates. Rhizosphere bacteria can be classified into three main groups: (1) beneficial bacteria that promote plant growth (PGPR—plant growth-promoting rhizobacteria), (2) phytopathogenic bacteria causing plant diseases, and (3) neutral bacteria [9]. PGPR that produce bacteriocins contribute to plant growth not only by competing with other microbes but also by decreasing pathogen populations in the root zone [16]. These bacteriocins represent environmentally friendly alternatives to conventional pesticides and chemicals. Their production is induced by stress and microbial interactions; increased bacterial density triggers quorum-sensing mechanisms that stimulate bacteriocin synthesis, thereby enhancing their competitive efficacy in plant protection [8].

An example of beneficial bacteriocin activity is thuricin 17, produced by *Bacillus thuringiensis* NEB17, which selectively targets specific strains without negatively affecting beneficial PGPR such as rhizobia, *Pseudomonas*, and other *Bacillus species*. This specificity is linked to the occupation of distinct ecological niches, for instance, rhizobia inhabit root nodules, whereas *Bacillus* species colonize the root cortex, reducing direct competition.

Moreover, co-inoculation of bacteriocin-producing bacteria (e.g., *Bacillus thuringiensis*) with nodule-forming bacteria (e.g., *Bradyrhizobium japonicum*) can enhance nodule formation, further stimulating plant growth. Bacteriocins exert their beneficial effects through multiple mechanisms: directly suppressing pathogens, boosting plant immune responses, and fostering favorable conditions for beneficial microbes. Consequently, bacteriocin production supports PGPR persistence in the rhizosphere and indirectly promotes plant development [16].

Moreover, the previously mentioned bacteriocin thuricin 17 exerts a significant growth-promoting effect on plants, particularly under stress conditions. Treatment with this compound activates plant defense responses, including enhanced synthesis of phenolic compounds, increased activity of lignification-related enzymes such as phenylalanine ammonia-lyase (PAL), and elevated activity of antioxidant enzymes, including peroxidase (POD) and superoxide dismutase (SOD). Furthermore, thuricin 17 acts as a pseudo-stress signal—i.e., a compound that mimics stress without causing damage—by binding to plant receptors and triggering metabolic pathways that enhance photosynthetic rate and overall growth even in the absence of actual stress. Application of thuricin 17 also promotes nodule formation, root biomass, and shoot biomass in soybean (*Glycine max*), while improving leaf area, leaf greenness, and nitrogen concentration. These effects are observed not only in dicotyledonous plants like soybeans but also in monocotyledonous plants such as maize *(Zea mays*), indicating broad-spectrum activity. Under stress conditions including drought, salinity, or extreme temperatures, thuricin 17 demonstrates particularly pronounced beneficial effects. For instance, in soybean, it mitigates the adverse effects of water deficit by increasing root and nodule biomass; in rapeseed (*Brassica napus*), it alleviates temperature and salt stress by stimulating root and leaf development [16].

Members of the genus Bacillus produce a wide range of PGPR metabolites. A review by Kloepper et al. [117] highlights the potential of this bacterial group as promising PGPR biopreparations for crop production. The authors documented the efficacy of these bacteria in protecting crops against Cercospora leaf spot of sugar beet (*Beta vulgaris*), bacterial leaf spot of radish (*Raphanus sativus*) caused by Xanthomonas campestris pv. armoraciae, cucumber mosaic virus, tobacco blue mold (*Peronospora tabacina*), cucurbit bacterial wilt (*Erwinia tracheiphila*), and tomato late blight (*Phytophthora infestans*), among others [117].

However, comprehensive studies are essential before incorporating bacteriocin-producing strains into multi-strain biofertilizers. This caution stems from the risk that bacteriocin-producing strains may suppress other beneficial microorganisms included in commercial products. For example, Hafeez et al. [118] demonstrated that a bacteriocin-producing strain of *Rhizobium leguminosarum* bv. *viciae* LC-31 inhibited the growth of *Rhizobium* sp. and *Agrobacterium* sp., which are commonly added as phosphate solubilizers in commercial fertilizers.

The efficacy of bacteriocin-producing strains must be validated in advance under controlled conditions. For instance, Rodelas et al. [119] assessed the activity of 20 *Rhizobium leguminosarum* bv. *viciae* strains isolated from five distinct agroecosystems in the Granada region (Spain). The study was performed under gnotobiotic conditions using faba bean (*Vicia faba* cultivar *Alborea*) as the host plant. Leonard jars were employed to evaluate strain efficacy based on nodulation capacity and nitrogenase activity.

Results identified strain Z25 as the most promising isolate, exhibiting high nodule dry matter accumulation and broad bacteriocinogenic activity against rhizobia, with strong potential to inhibit other strains. Strain Z25 was confirmed to produce proteinaceous antimicrobial bacteriocins under various cultivation conditions. These findings suggest the potential of strain Z25 as a bacterial inoculant in agricultural applications [119].

### 3.4. Bacteriocin Applications in Food Manufacturing

In food manufacturing, bacteriocins are applied via several strategies: purified bacteriocins, preparations containing bacteriocins derived from fermentation processes, and cultures of bacteriocin-producing strains [10]. Bacteriocins are typically colorless, odorless, and tasteless, which further enhances their applicability [120].

Bacteriocins produced by lactic acid bacteria are of particular importance for applications related to the human body. It is crucial to note that while many LAB species have generally recognized as safe (GRAS) status; this status for antimicrobial peptides is substance-specific. To date, only specific bacteriocins—particularly nisin and pediocin PA-1—have obtained official GRAS status from the FDA for use in food products. Indeed, the first widely utilized bacteriocins (nisin and pediocin PA-1) originated from LAB [69].

However, the antimicrobial activity of LAB bacteriocins is variable and influenced by the chemical composition and physical conditions of the food matrix, including pH, temperature, and the presence of proteases, peptidases, and NaCl. Optimal bacteriocin production occurs at approximately pH 5.5, which differs from the optimal growth pH for LAB (~pH 6.5). Nisin demonstrates greater stability and activity in acidic food products (pH < 7). NaCl can inhibit LAB growth and bacteriocin production while simultaneously protecting certain pathogens, such as *L. monocytogenes*, from bacteriocin action. Elevated temperatures and proteolysis (e.g., during cheese ripening) reduce bacteriocin activity. Although minimum inhibitory concentration (MIC) and minimum bactericidal concentration (MBC) values are determined using standardized cell counts to quantify the intrinsic activity of a bacteriocin, its practical efficacy in applied settings, such as food systems, can depend on the initial pathogen load. Higher contamination levels may require increased bacteriocin concentrations to achieve effective control, as they can exceed the capacity of a given dose to inhibit or eradicate the entire population [4].

Lacticin 3147 from *Lactococcus lactis* demonstrates broad and potent antimicrobial potential against Gram-positive bacteria (*L. monocytogenes*, *Staphylococcus aureus*, *Bacillus cereus*, and *Clostridium perfringens*), with a spectrum comparable to that of nisin. Its lack of activity against Gram-negative bacteria confirms its specificity. The promising biopreservative potential of lacticin 3147 has been confirmed in numerous studies using diverse food matrices. The preparation has been applied both as an exogenously produced bacteriocin and in situ by producer strains. An important aspect is the synergistic effect of lacticin 3147 when combined with other hurdle technologies. For instance, its combination with high hydrostatic pressure reduced pathogen levels in milk and whey. Similarly, combination with organic acids enhanced antimicrobial activity in meat product models, underscoring the practical value of integrated biopreservation approaches [52].

## 4. Industrial Production of Bacteriocins

Realizing the full potential of bacteriocins requires the development and optimization of cost-effective biotechnological platforms dedicated to the efficient production, isolation, and purification of recombinant antimicrobial peptides expressed by targeted producer strains [68].

The industrial production of bacteriocins for commercial use involves a multi-stage biotechnological process optimized for maximum cost-effectiveness and quality standardization. The primary method for large-scale production remains fermentation in bioreactors. Producer strains are cultivated in nutrient media, often incorporating food industry byproducts such as whey, corn steep liquor, or molasses to ensure cost efficiency. Critical physicochemical parameters, including temperature, pH, dissolved oxygen concentration, and agitation rate, are maintained within strictly defined limits using automated control systems. Subsequent downstream processing focuses on the isolation, concentration, and stabilization of bacteriocins.

For the application of purified bacteriocins, efficient and cost-effective methods for isolation and purification are required. Purification methods often rely on phase separation and adsorption/desorption principles. Concentration via precipitation (e.g., with ammonium sulfate as a salting-out agent) is frequently employed prior to purification to enhance yield; the resultant protein precipitate demonstrates resistance to bacterial and proteolytic degradation. Alternative precipitants include acetone, ethanol, or polyethylene glycol, though ammonium sulfate precipitation remains economically advantageous due to its accessibility. Gel filtration chromatography, which exploits molecular weight differences, is commonly used as a subsequent purification step. Native bacteriocin-producing strains typically yield low quantities of target compounds, underscoring the potential of genetic engineering to enhance production [18].

The utility of bacteriocins across various sectors is further enhanced by their stability under diverse physicochemical conditions. For example, thuricin 17 exhibits high resistance to denaturation across a temperature range from −20 °C to 100 °C and maintains biological stability within a pH range of 1.0 to 9.25 [16]. Gupta and Pandey [121] reported that bacteriocins from several producer strains retained remarkable antagonistic activity against *Bacillus subtilis*, *Staphylococcus aureus*, *E. coli*, and *Pseudomonas aeruginosa* after exposure to high temperature (121 °C), high salt concentration (10%), and a wide pH range (3 to 9) [29]. Another study showed that a low-molecular-weight bacteriocin produced by *Lactiplantibacillus plantarum* tolerated a broad pH range (2–10), temperatures (−20 to 120 °C), and high NaCl concentrations. It should be noted that the analyzed bacteriocin retained its activity even after storage for 6 months at low temperature (4 °C) in vacuum-sealed packaging [122].

The significant challenge for bacteriocin industrial production is the low production yield and limited spectrum of activity of many native bacteriocins. This creates substantial opportunities for bioengineering and synthetic biology approaches. Future efforts should focus on genetic modification of producer strains to enhance bacteriocin synthesis and secretion. Furthermore, protein engineering methods can be employed to develop novel bacteriocin analogs with improved characteristics, such as enhanced stability under physiological conditions, broader antimicrobial spectra (including activity against Gram-negative pathogens), and reduced potential for resistance development. The creation of such “tailored” bacteriocins represents a new frontier in developing highly specific and effective tools for sustainable agriculture.

Although still limited, commercial products containing semi-purified or pure bacteriocins are available and have industrial applications. Table 4 presents examples of commercial bacteriocin-containing products used across various sectors, including the food industry, agriculture, and veterinary medicine. The widespread use of nisin deserves special attention. For instance, Nisaplin^®^, containing 2.5% nisin by weight, is employed to inhibit the growth of pathogenic microorganisms in dairy products, pasteurized juices, and canned vegetables.

Veterinary applications of bacteriocins are particularly noteworthy. Products such as Re-Tain^®^ and Wipe Out^®^ are designed to treat subclinical mastitis in dairy cattle. Their efficacy is validated by a significant reduction in pathogen loads, including *Staphylococcus aureus* and *Streptococcus agalactiae*, positioning them as promising alternatives to conventional antibiotics.

Thus, the commercial products summarized in Table 4 underscore the practical significance of bacteriocins and suggest substantial promise for broader implementation across the industrial and agricultural biotechnology sectors.

## 5. Limitations of Bacteriocin Usage

### 5.1. Safety of Bacteriocins

Widespread adoption of bacteriocins in agricultural production faces several obstacles requiring close attention. A critical factor is the assessment of their toxicity and safety profiles.

While bacteriocins are generally considered non-toxic to eukaryotic cells and hold GRAS status [19], their amino acid sequence specificity necessitates rigorous cytotoxicity evaluations as an integral part of their safety profile. Their selective activity against prokaryotic cells underpins their suitability for agricultural applications, ensuring safety for plants, livestock, and consumers when used as alternatives to conventional antibiotics. Systematic evaluation of cytotoxicity, immunogenicity, and impact on gut microbiomes and the environment is crucial for regulatory approval and public acceptance.

Among the diverse bacteriocins identified, only nisin and pediocin PA-1, produced by Gram-positive bacteria with GRAS status, are GRAS for human consumption. Microcins are produced by *E. coli* strains and other *Enterobacteriaceae* such as *Klebsiella pneumoniae*, which are not considered as GRAS microorganisms; however, antimicrobial peptides can be delivered as cell-free preparations and their antimicrobial activity, predominantly restricted to Gram-negative bacteria, can be explored [2].

### 5.2. Development of Resistance to Bacteriocins

Bacteriocin efficacy can be compromised by the development of pathogen resistance. This resistance may be innate (intrinsic to certain species) or acquired (emerging in initially susceptible strains). Although a standardized definition of resistance is lacking in the literature, it is typically assessed based on bacterial growth in the presence of high bacteriocin concentrations. Environmental factors such as pH or salt concentration can partially mitigate resistance development [19].

Resistance to bacteriocins can be innate or acquired and arises through multiple mechanisms. Specific molecular mechanisms include alteration of bacteriocin receptors such as the mannose-specific phosphotransferase system (Man-PTS), which some bacteriocins use to form pores in target cells; chemical modifications of cell surface components like teichoic acids and lipopolysaccharides that reduce bacteriocin binding; production of capsules to prevent bacteriocin contact with the cell surface; enzymatic inactivation of bacteriocins by secreted peptidases; and active efflux of bacteriocins using efflux pumps [130,131].

Resistance development frequency varies widely, influenced by bacterial strain, bacteriocin type, environmental factors, and culture conditions. Some studies have shown resistance frequencies from as low as 10^−9^ to as high as 10^−2^. For certain Class IIa bacteriocins, high-level resistance in bacteria like *L. monocytogenes* is associated with a prevalent general mechanism of cell envelope modification [130,132].

Moreover, resistance to bacteriocins can sometimes be linked to cross-resistance or co-resistance with antibiotics, meaning bacteria resistant to bacteriocins may also display altered antibiotic susceptibility.

## 6. Conclusions

Bacteriocins represent a promising and sustainable alternative to traditional antibacterial agents in primary food production systems due to their high specificity, safety for humans and animals, and minimal environmental impact. This review synthesizes key evidence for bacteriocin applications across livestock farming, poultry production, aquaculture, crop protection, and food manufacturing.

Major findings demonstrate that established bacteriocins, particularly nisin and pediocin PA-1 (both with GRAS status), exhibit broad-spectrum activity against critical agricultural pathogens. In livestock farming, nisin achieves up to 90% cure rates for mastitis caused by *Staphylococcus aureus* and *Streptococcus agalactiae*, with commercial products (Wipe Out, Mast Out) already approved for veterinary use. In poultry farming, bacteriocins effectively reduce intestinal colonization by *Clostridium perfringens* and *Escherichia coli*, decreasing liver pathogen recovery from 60% to 8% and reducing *Campylobacter* counts by 10^5^–10^6^-fold pre-slaughter.

In aquaculture, bacteriocins show significant potential for controlling major pathogens affecting fish and crustaceans. Well-characterized bacteriocins including enterocin AS-48, mundticin KS, nisin Z, and pediocin PA-1 demonstrate confirmed efficacy against *Lactococcus garvieae*, *Aeromonas hydrophila*, *Vibrio parahaemolyticus*, *Vibrio alginolyticus*, *Pseudomonas aeruginosa*, *Shewanella putrefaciens*, and *Listeria monocytogenes* in multiple aquatic species (salmon, shrimp, tilapia, catfish). Application strategies include both live bacteriocin-producing probiotic strains administered via feed or water, and purified bacteriocin preparations incorporated into feed pellets. This dual approach enables flexible implementation depending on cultivation system, target pathogen, and cost-effectiveness considerations. The rapid growth of aquaculture production (projected 109 million tons by 2030) underscores the critical importance of bacteriocin-based alternatives to reduce antibiotic dependence and resistance development in aquatic environments.

In crop production, bacteriocins from plant growth-promoting rhizobacteria (PGPR) not only suppress phytopathogens such as *Xanthomonas* spp., *Erwinia* spp., and *Pseudomonas* spp., but also stimulate plant growth under stress conditions. Commercial bacteriocin products (e.g., Nisaplin, containing 2.5% nisin) are successfully employed in food preservation, inhibiting pathogenic microorganisms in dairy products, juices, and canned vegetables.

Critical challenges limiting broader bacteriocin adoption include (1) high production and purification costs compared to conventional antibiotics; (2) potential resistance development through receptor modification and cell envelope changes; (3) regulatory barriers requiring extensive safety and efficacy documentation; and (4) stability challenges under diverse application conditions. Among diverse bacteriocins identified, only nisin and pediocin PA-1 have achieved GRAS status for food applications.

Future research priorities must focus on (1) optimizing cost-effective production and purification methods, including genetic engineering to enhance yields; (2) developing combination therapies and bacteriocin cocktails targeting multiple cellular pathways to prevent resistance; (3) advancing formulation technologies such as microencapsulation for improved stability and delivery; (4) establishing clear regulatory frameworks for bacteriocin use across agricultural sectors; and (5) expanding the structural and functional characterization of promising but poorly defined bacteriocin-like substances from environmental isolates.

In the context of escalating antibiotic resistance and environmental sustainability imperatives, bacteriocins offer scientifically validated, commercially feasible solutions for managing plant and animal health across agriculture. Their targeted antimicrobial action, combined with minimal disruption to beneficial microbiota and rapid degradation in the environment, positions bacteriocins as key components of integrated biosecurity strategies for sustainable agro-industrial systems. Successful implementation will require interdisciplinary collaboration encompassing microbiology, genetic engineering, formulation science, regulatory affairs, and agricultural economics.

## Figures and Tables

**Table 1:** Diversity of bacteriocins identified from certain *Bacillus* strains [2,29].

Strain Name	Identified Bacteriocin	Molecular Weight of Bacteriocin, kDa	Chemical Family/Type	References
Thuricins				
*Bacillus thuringiensis* HD-2	Thuricin	>950	Ribosomally synthesized and post-translationally modified peptides (RiPPs)	[30]
*Bacillus thuringiensis* serovar huazhongensis	Thuricin Z	~3.6	Class I—Sactibiotics	[31]
*Bacillus thuringiensis* LX43	Thuricins A1, A2, A3, A4, A5	~3–4	Leaderless unmodified bacteriocins	[32]
*Bacillus thuringiensis* BMG1.7	Thuricin 7 *	11.6	Class IId—Non-pediocin-like bacteriocins	[33]
*Bacillus thuringiensis* B439	Thuricin 439 *	3	Class I—Sactibiotics (two-component sactipeptide)	[34]
*Bacillus thuringiensis* NEB17	Thuricin 17	3.16	Class IId—Non-pediocin-like bacteriocins	[35]
*Bacillus thuringiensis* subsp. *entomocidus* HD198	Thuricin S	3.1	Class IId—Non-pediocin-like bacteriocins	[36]
*Bacillus thuringiensis* SF361	Thuricin H	3.1	Class IId—Non-pediocin-like bacteriocins	[37]
*Bacillus thuringiensis* DPC6431	Thuricin CD	2.762/2.861	Class I—Sactibiotics	[38]
Entomocins				
*Bacillus thuringiensis* subsp. *entomocidus* HD9	Entomocin 9	12.4	Class II (intermediate-size peptide)	[39]
*Bacillus thuringiensis* subsp. *entomocidus* HD110	Entomocin 110	4.8	Class II (small peptide)	[40]
*Bacillus thuringiensis* subsp. *entomocidus* LBIT 420	Entomocin 420	10	Class II—BLIS (bacteriocin-like inhibitory substances)	[41]
Other bacteriocins				
*Bacillus thuringiensis* subsp. *tochigiensis* HD868	Tochicin	10.5	Class II—Intermediate-size peptide	[42]
*Bacillus thuringiensis* BUPM4	Bacthuricin F4	3.1	Class IId—Non-pediocin-like bacteriocins (thuricin-like)	[43]
*Bacillus thuringiensis* subsp. *morrisoni* LBIT 269	Morricin 269	10	Class II—BLIS (bacteriocin-like inhibitory substances)	[44,45]
*Bacillus thuringiensis* subsp. *kurstaki* LBIT 287	Kurstacin 287	10	Class II—BLIS (bacteriocin-like inhibitory substances)	[44,45]
*Bacillus thuringiensis* subsp. *kenyae* LBIT 404	Kenyacin 404	10	Class II—BLIS (bacteriocin-like inhibitory substances)	[44,45]
*Bacillus thuringiensis* subsp. *tolworthi* LBIT 524	Tolworthcin 524	10	Class IId—Thuricin-like peptides	[44,45]
*Bacillus thuringiensis* subsp*. kurstaki* HD-1	Bn1	3.193	Class IId—Thuricin-like bacteriocins	[46]
*Lactococcus lactis subsp. lactis* DPC3147	Lacticin 3147	3.3, between 10 and 25, 6.11	Class I—Lantibiotics (two-component lantibiotic)	[47]
*Bacillus amyloliquefaciens*, *Bacillus pumilus*	Plantazolicin	1.354	Class I—Linear azole-containing peptides (LAPs)	[48]
*Bacillus coagulans*	Circularin A	–	Class V/IIc—Circular bacteriocins (Subgroup I)	[49]
*Bacillus paralicheniformis*	LichenicidinVK21A2	–	Class I—Lantibiotics (two-component lantibiotic)	[50]
*Bacillus subtilis*	Subtilosin A	3.4	Class I—Sactibiotics	[51]

– = the molecular weight was not reported. * Some bacteriocins (thuricin 7, thuricin 439) are poorly or preliminarily characterized. Their exact structure and classification require further investigation. These may be identical to other previously described bacteriocins or could belong to different chemical groups upon complete characterization.

**Table 3:** Antimicrobial spectrum of bacteriocins against aquaculture pathogens (adapted from reference [76] with additional data from references [80,81,82,83,84,85,86,87,88,89,90,91,92,93]).

Bacteriocin Tested	Inhibited Bacterial Pathogens	Aquaculture Species	Reference
Well-characterized bacteriocins			
Enterocin AS-48 produced by *Enterococcus* strains	*Lactococcus garvieae*	*Oncorhynchus tshawytscha*	[80]
Mundticin KS produced by *Enterococcus mundtii* NFRI 7393	*Pseudomonas aeruginosa*, *Shewanella putrefaciens*	*Odontesthes platensis*	[81]
Nisin Z produced by *Lactococcus lactis*	*Lactococcus garvieae*	*Odontesthes platensis*	[82]
Nisin produced by *Lactococcus lactis* (2.5% balance sodium chloride, Sigma-Aldrich, Singapore)	*L. monocytogenes*	*Litopenaeus vannamei*	[83]
Bacteriocin isolated from *Pediococcus acidilactici*	*L. monocytogenes*	*Tilapia* sp., *Catla catla*, *Cyprinus carpio*	[84]
Plantaricin LPL-1 produced by *Lactiplantibacillus plantarum* LPL-1	*L. monocytogenes*	Acipenseridae, *Oncorhynchus clarkii*	[85]
Partially and poorly characterized substances			
CAMT2 produced by *Bacillus amyloliquefaciens* ZJHD3-06	*L. monocytogenes*, *Staphylococcus aureus*	*Epinephelus areolatus*	[86]
Bacteriocin produced by *Bacillus subtilis* LR1	*Aeromonas hydrophila*, *Aeromonas salmonicida*, *Bacillus mycoides*, *Pseudomonas fluorescens*	*Labeo rohita*	[87]
Bacteriocin 99% homologous to that produced by *Bacillus* sp.	*Vibrio alginolyticus*, *Aeromonas hydrophila*, *Pseudomonas stutzeri*	*Penaeus monodon*	[88]
Coagulin L1208 produced by *Bacillus* coagulans L1208	*E. coli*, *Shewanella putrefaciens*, *Staphylococcus aureus*	*Pseudosciaena croce*	[89]
Bacteriocin produced by *Lactiplantibacillus plantarum* FGC-12	*Vibrio parahaemolyticus*	*Litopenaeus vannamei*	[90]
PSY2 produced by *Lactococcus lactis* PSY2	*L. monocytogenes*	*Perca* sp., *Tuna* sp., *Platax* sp.	[91]
7293 produced by *Weissella hellenica* BCC 7293	*L. monocytogenes*, *Staphylococcus aureus*, *Aeromonas hydrophila*, *E. coli*, *Pseudomonas aeruginosa*, *Salmonella typhimurium*	*Pangasius bocourti*	[92]
PE-ZYB1 produced by *Pediococcus pentosaceus* zy-B	*L. monocytogenes*	*Mimachlamys nobilis*	[93]

**Table 4:** Selected commercial bacteriocin-containing products.

Commercial Designation	Manufacturer	Composition	Field of Application	Reference
Nisaplin^®^	Danisco, Copenhagen, Denmark; Paris, France	2.5% nisin, NaCl, and milk-derived protein solids	Food industry: soft cheeses, cottage cheese, processed cheese, milk and dairy desserts (yogurt, sour cream, pudding), pasteurized juices, mayonnaise, sauces, canned vegetable products	[79]
Preva Medicated Wipes	Bayer HealthCare, Leverkusen, Germany	Nisin	Pet wipes for dermal applications: management of bacterial skin infections, general skin/coat hygiene	[123]
Wipe Out^®^ Dairy Wipes	ImmuCell Corporation, Portland, ME, USA	Nisin	Pre-milking teat sanitization in dairy cows	[124]
Mast Out^®^	ImmuCell Corporation, Portland, ME, USA	Nisin	Intramammary treatment of mastitis in lactating dairy cows	[125]
Nisin Z^®^P	Handary, Brussels, Belgium	Purified nisin (>95%)	Anti-staphylococcal mastitis formulations; anti-pathogen agent in toothpaste and skincare products	[126,127]
NisinA^®^	Handary, Brussels, Belgium	Nisin A (fermentation-derived)	Processed foods: shelf-life extension via suppression of Gram-positive bacteria	[124]
NisinZ^®^	Handary, Brussels, Belgium	Food-grade nisin Z concentrate (fermentation-derived)	Processed foods: shelf-life extension via suppression of Gram-positive bacteria	[127]
White NisinA^®^	Handary, Brussels, Belgium	Clarified nisin concentrate (fermentation-derived)	Prevention of microbial spoilage and color fading in juice-based beverages	[127]
NisinA^®^ P	Handary, Brussels, Belgium	Ultra-pure nisin A concentrate (fermentation-derived)	Healthcare: anti-staphylococcal mastitis formulations; anti-pathogen agent in oral hygiene and dermatological products	[127]
Microgard^®^ 100, 210, 400, 430, 730, 740	Danisco, Copenhagen, Denmark, Paris, France	Pediocin PA-1	Antimicrobial fermentates with antifungal activity and Gram-negative bacterial inhibition	[128]
BioSafe^®^	Chr. Hansen, Hørsholm, Denmark	*Pediococcus acidilactici* (sakacin A producer), *Latilactobacillus curvatus* (pediocin PA-1/AcH producer) and *Staphylococcus xylosus*	Control of *L. monocytogenes* dissemination in food production systems	[128,129]
Bactoferm^®^	Chr. Hansen, Hørsholm, Denmark	Pediocin—and sakacin-producing strains	Manufacturing fermented sausages and dry-cured meats	[115]
HOLDBAC^®^	Dupont Nutrition Biosciences Aps, Copenhagen, Denmark	Bacteriocin-producing consortium: *Propionibacterium freudenreichii* subsp. *shermanii* DSM 706 + *Limosilactobacillus rhamnosus* DSM 7061	Seafood, meat, and dairy preservation against *Listeria*, yeasts, and molds	[10,129]

## Data Availability

No new data were created.

## Abbreviations

BLIS	Bacteriocin-like inhibitory substances
ESFA	European Food Safety Authority
FAO	Food and Agriculture Organization of the United Nations
FDA	Food and Drug Administration
GIT	Gastrointestinal tract
GRAS	Generally Recognized As Safe
LAB	Lactic acid bacteria
MV	Measles virus
MIC	Minimum inhibitory concentration
MBC	Minimum bactericidal concentration
PGPR	Plant growth-promoting rhizobacteria
WHO	World Health Organization
